# Multiple Measures of Mineral Metabolism Were Associated With Renal Function in Chinese Centenarians: A Cross-Sectional Study

**DOI:** 10.3389/fmed.2020.00120

**Published:** 2020-04-15

**Authors:** Shihui Fu, Haotian Yu, Yulong Li, Faqin Lv, Juelin Deng, Fu Zhang, Fuxin Luan, Yali Zhao, Yao Yao

**Affiliations:** ^1^Department of Geriatric Cardiology, Chinese People's Liberation Army General Hospital, Beijing, China; ^2^Department of Cardiology, Hainan Hospital of Chinese People's Liberation Army General Hospital, Sanya, China; ^3^Central Laboratory, Hainan Hospital of Chinese People's Liberation Army General Hospital, Sanya, China; ^4^Department of Ultrasound, Hainan Hospital of Chinese People's Liberation Army General Hospital, Sanya, China; ^5^Center for the Study of Aging and Human Development and Geriatrics Division, Medical School of Duke University, Durham, NC, United States; ^6^Center for Healthy Ageing and Development Studies, National School of Development, Peking University, Beijing, China

**Keywords:** Chinese centenarians, mineral metabolism, parathyroid hormone, total procollagen type 1 N-terminal propeptide, estimated glomerular filtration rate

## Abstract

**Background:** Chronic kidney disease (CKD) constitutes a public health issue that is estimated to affect more than 10% of global population. Over 100 million people have CKD in China. For the first time, this cross-sectional study was designed to determine whether multiple measures of mineral metabolism had a significant association with renal function in Chinese centenarians.

**Methods:** China Hainan Centenarian Cohort Study was conducted in 18 cities and counties of Hainan Province, China. It utilized the standard methodology for home interview and blood analyses in 750 centenarians including 608 females and 142 males.

**Results:** All centenarians have a median (range) age of 102 (100–115) years, and median (range) level of estimated glomerular filtration rate (eGFR) was 63 (16–138) ml/min/1.73 m^2^. There were 318 centenarians (42.4%) with eGFR levels <60 ml/min/1.73 m^2^. In simple correlation and multivariate linear regression analyses, serum phosphorus, osteocalcin, β-crosslaps, total procollagen type 1 N-terminal propeptide (TP1NP), and parathyroid hormone (PTH) levels were negatively associated with eGFR levels (*P* < 0.05 for all).

**Conclusion:** The current study supported that serum phosphorus, osteocalcin, β-crosslaps, TP1NP, and PTH levels were negatively associated with eGFR levels, and demonstrated a significant association between multiple measures of mineral metabolism and renal function in Chinese centenarians.

## Background

Chronic kidney disease (CKD) constitutes a public health issue that is estimated to affect more than 10% of global population, and over 100 million people have CKD in China (1). CKD not only has an increased prevalence in recent years, but also is significantly associated with poor outcome (2, 3). Meanwhile, mineral metabolism has been suggested to be significantly implicated in the rapid progression of CKD (4–6). Previous studies exploring the association between mineral metabolism and renal function have been conducted in the CKD population, among general adults and within the elderly, but nearly no report has been published for centenarians, especially in China (7, 8). Significant differences in the serum parathyroid hormone (PTH) levels, but not serum calcium and 25-hydroxyvitamin D (25OHD) levels, have been identified within different estimated glomerular filtration rate (eGFR) levels (7). Another study has found insignificant correlation between serum 25OHD and eGFR levels (8).

Centenarians have been suggested to have a delayed or escaped onset and interaction of age-related illnesses, such as metabolic bone disease (MBD) and CKD (9, 10). Some centenarians may experience a delayed onset of age-related illnesses (delayers), while others may not succumb to any age-related illnesses (escapers) (9, 11). Thus, centenarians may represent a prototype of successful aging (9, 12). More importantly, what is this model of centenarian longevity and successful aging? Studies analyzing this model in the centenarians could provide valuable information for an early prevention of age-related diseases and the promotion of aging successfully (13). As a possible part of this model, whether the interaction between mineral metabolism and renal function exists in the aging process of centenarians is still unclear and needs to be studied further (9).

Meanwhile, considering a lack of the data regarding centenarians, there is a need of studies evaluating the association between mineral metabolism and renal function in Chinese centenarians. Hainan is a longevity area with the highest population density of centenarians in China. The China Hainan Centenarian Cohort Study (CHCCS) provides a considerably population-based sample of Chinese centenarians (14). Moreover, in order to better understand the association and promote the development of medical tourism and health technology of Hainan, China,it is very important to first establish the association between mineral metabolism and renal function in the untreated state. For the first time, the aim of this cross-sectional study was to determine whether multiple measures of mineral metabolism had a significant association with renal function in Chinese centenarians who were not receiving vitamin D and phosphate binder.

## Methods

### Study Population

CHCCS was a population-based study conducted in 18 cities and counties of Hainan Province, China, between July 2014 and December 2016. Its cohort profile has been described previously (15). According to the National Civil Registry, 1,002 centenarians (≥100 years) were identified by Hainan Civil Affairs Bureau and enrolled in the current study (16). Demographic information (age and sex) were registered and ascertained from national identification cards. Inclusion criteria: (1) was 100 years or older; (2) volunteered to participate in the study and provided written informed consent; and (3) was conscious and could cooperate to complete the home interview, physical examination, and blood analyses (16). Exclusion criteria: (1) personal identity information was not complete or identification cards showed an age of <100 years; (2) refused to comply with the requirements of the study, including the collection of blood samples (16). There were 750 centenarians included in the final analyses. All centenarians did not receive active or nutritional vitamin D and phosphate binder. All procedures performed in studies involving human participants were in accordance with the ethical standards of Hainan branch of Chinese People's Liberation Army General Hospital (Sanya, Hainan; Number: 301hn11201601) and with the 1964 Helsinki declaration and its later amendments or comparable ethical standards. Informed consent was obtained from all participants included in the study.

### Standard Process

This cross-sectional study utilized the standard methodology for home interview, physical examination and blood analyses. There were internists, geriatricians, cardiologists, endocrinologists, nephrologists, and nurses in the research team (14). Waist circumference was measured with a soft tape midway between the lowest rib and the iliac crest. Consistent with current recommendations, systolic and diastolic blood pressures (SBP and DBP) were measured with the right arm of centenarians two times consecutively, with at least 1 min between measurements, and the reported blood pressures were the average of two measurements (9). Within 4 h, all blood samples were received from each centenarian, transported in a chilled bio-transport container (4°C) and analyzed at one Central Laboratory. Serum triglyceride, low-density lipoprotein cholesterol (LDL-C), high-density lipoproteincholesterol (HDL-C), fasting blood glucose (FBG), and creatinine levels were determined with enzymatic assays (Roche Products Ltd, Basel, Switzerland) on a fully automatic biochemical analyzer (Cobas c702; Roche Products Ltd, Basel, Switzerland). Serum PTH levels were determined with automatic electrochemiluminescence immunoassay (ECLIA) analyzers (Cobas e602; Roche Products Ltd, Basel, Switzerland). Serum 25OHD levels were determined with automated radioimmunoassay analyzers (DiaSorin, Stillwater, MN, USA) to reflect vitamin D status (17). eGFR levels were determined with a modified version of Modification of Diet in Renal Disease (MDRD) equation based on the data from Chinese population as follows: 175 × serum creatinine (mg/dL)^−1.234^ × age (year)^−0.179^ × 0.79 (if female) (18). Diabetes mellitus was defined as FBG ≥7.0 mmol/L or taking hypoglycemic drugs/insulin. Hypertension was defined as SBP ≥140 mmHg, DBP ≥90 mmHg or taking anti-hypertensive drugs (9).

### Statistical Analyses

Continuous variables with skewed distribution were reported as medians and interquartile ranges, and compared with Mann–Whitney *U*-test. Categorical variables were reported as numbers and percentages, and compared with Chi-square test. Spearman's correlation (continuous variables with skewed distribution and categorical variables) was used to investigate the simple correlation between mineral metabolism and renal function. Linear regression analysis was conducted following these models: model 1 adjusted for age and sex; and model 2 adjusted for age, sex, waist circumference, hypertension, diabetes mellitus, SBP, DBP, serum albumin, triglyceride, HDL-C, LDL-C, and FBG levels, so as to investigate the association between mineral metabolism and renal function. Besides, linear regression analysis was conducted following these models: model 1 adjusted for age, sex, and eGFR levels; and model 2 adjusted for age, sex, waist circumference, hypertension, diabetes mellitus, SBP, DBP, serum albumin, triglyceride, HDL-C, LDL-C, FBG, and eGFR levels, so as to investigate the association between serum PTH and 25OHD levels additionally adjusted for eGFR levels. Unstandardized β were provided and shown per 1-SD increment. As the common statistics in linear regression analyses, they were automatically and simultaneously output by Statistic Package for Social Science (SPSS) software. Statistical analyses were conducted with SPSS software version 17 (SPSS Inc., Chicago, IL, U.S.), with *P* < 0.05 considered to be statistically significant.

## Results

All centenarians have a median (range) age of 102 (100–115) years, and 18.9% of these centenarians were males. Median (range) level of eGFR was 63 (16–138) ml/min/1.73 m^2^. There were 318 centenarians (42.4%) with eGFR levels <60 ml/min/1.73 m^2^. Table 1 displays the characteristics of these centenarians. Serum osteocalcin, β-crosslaps, TP1NP, and PTH levels (*P* < 0.05 for all) rather than serum calcium and 25OHD levels (*P* > 0.05 for all) had significant difference between centenarians with eGFR levels <60 ml/min/1.73 m^2^ and those with eGFR levels ≥60 ml/min/1.73 m^2^. In the simple correlation analyses, serum phosphorus, osteocalcin, β-crosslaps, TP1NP, and PTH levels were negatively related to eGFR levels (*P* < 0.05 for all), whereas serum calcium and 25OHD levels were not significantly related to eGFR levels (*P* > 0.05 for all). In the first and second models of linear regression analyses (Table 2), serum phosphorus, osteocalcin, β-crosslaps, TP1NP, and PTH levels were negatively associated with eGFR levels (*P* < 0.05 for all), whereas serum calcium and 25OHD levels were not significantly associated with eGFR levels (*P* > 0.05 for all). Serum 25OHD levels were significantly associated with serum PTH levels in the first and second models of linear regression analyses after adjusted for eGFR levels (*P* < 0.05 for all).

**Table 1:** Characteristics of all 750 centenarians divided by eGFR levels and their correlations with eGFR levels in simple correlation analyses.

**Characteristics**	**All (*n* = 750)**	**eGFR <60 ml/min/1.73 m^**2**^ (*n* = 318)^a^**	**eGFR ≥60 ml/min/1.73 m^**2**^ (*n* = 432)^a^**
Age (year)	102 (101–104)	102 (101–104)	102 (101–104)
Females (%)	608 (81.1)	249 (78.3)	359 (83.1)
Rural (%)	694 (92.5)	292 (91.8)	402 (93.1)
Unmarried (%)	4 (0.5)	2 (0.6)	2 (0.5)
Illiteracy (%)	682 (90.9)	283 (89.0)	399 (92.4)
Unknown income (%)	397 (52.9)	159 (50.0)	238 (55.1)
Smokers (%)	24 (3.2)	19 (6.0)	5 (1.2)^b^
Diabetes mellitus (%)	81 (10.8)	32 (10.1)	49 (11.3)
Hypertension (%)	551 (73.5)	245 (77.0)	306 (70.8)
WC (cm)	74 (69–80)	76 (71–82)	74 (68–79)^b^
SBP (mmHg)	150 (135–169)	152 (138–173)	148 (132–165)^b^
DBP (mmHg)	75 (67–83)	74 (67–85)	76 (67–82)
Albumin (g/L)	38.5 (35.8–41.3)	38.3 (35.4–41.2)	38.7 (35.9–41.4)
Phosphorus (mmol/L)	1.06 (0.94–1.16)	1.06 (0.95–1.18)	1.05 (0.94–1.16)
Calcium (mmol/L)	2.21 (2.13–2.28)	2.22 (2.13–2.29)	2.21 (2.13–2.28)
Osteocalcin (ng/ml)	29.42 (20.80–40.78)	34.47 (24.38–47.22)	26.76 (19.20–37.54)^b^
β-crosslaps (ng/ml)	0.41 (0.26–0.58)	0.45 (0.31–0.62)	0.39 (0.23–0.56)^b^
TP1NP (ug/L)	65.00 (47.00–91.00)	71 (52–99)	62 (44–86)^b^
PTH (pg/ml)	41.14 (33.28–61.33)	47.69 (35.39–63.94)	44.35 (31.83–58.42)^b^
25OHD (ng/ml)	21.50 (16.18–27.73)	21.20 (16.25–27.00)	21.60 (16.13–28.18)
Triglyceride (mmol/L)	1.04 (0.80–1.40)	1.14 (0.85–1.49)	0.99 (0.77–1.32)^b^
HDL-C (mmol/L)	1.39 (1.16–1.66)	1.35 (1.13–1.61)	1.42 (1.19–1.69)^b^
LDL-C (mmol/L)	2.72 (2.28–3.26)	2.65 (2.25–3.28)	2.74 (2.29–3.25)
FBG (mmol/L)	4.86 (4.25–5.77)	5.00 (4.26–5.78)	4.79 (4.22–5.76)

*eGFR, estimated glomerular filtration rate; WC, waist circumference; SBP, systolic blood pressure; DBP, diastolic blood pressure; TP1NP, total procollagen type 1 N-terminal propeptide; PTH: parathyroid hormone; 25OHD, 25-hydroxyvitamin D; TG, triglyceride; HDL-C, high-density lipoprotein-cholesterol; LDL-C, low-density lipoprotein-cholesterol; FBG, fasting blood glucose*.

a *All centenarians were categorized into centenarians with eGFR <60 ml/min/1.73 m^2^ and centenarians with eGFR ≥60 ml/min/1.73 m^2^*;

b *P < 0.05 computed from the comparison of characteristics between centenarians with eGFR <60 ml/min/1.73 m^2^ and centenarians with eGFR ≥60 ml/min/1.73 m^2^*.

**Table 2:** Association between parameters of mineral metabolism and eGFR levels in linear regression analyses of all 750 centenarians.

**Parameters**	**Models^a^**	**Unstandardized β^b^**	**95% confidence interval**
Phosphorus (mmol/L)	1st	−0.122	−0.176 to −0.067^c^
	2nd	−0.136	−0.190 to −0.081^c^
Calcium (mmol/L)	1st	−0.007	−0.086 to 0.072
	2nd	−0.006	−0.103 to 0.090
Osteocalcin (ng/ml)	1st	−0.002	−0.002 to −0.002^c^
	2nd	−0.002	−0.003 to −0.002^c^
β-crosslaps (ng/ml)	1st	−0.106	−0.137 to −0.075^c^
	2nd	−0.126	−0.156 to −0.095^c^
TP1NP (ug/L)	1st	0.000	0.000–0.000^c^
	2nd	0.000	0.000–0.000^c^
PTH (pg/ml)	1st	0.000	−0.001 to 0.000^c^
	2nd	0.000	−0.001 to 0.000^c^
25OHD (ng/ml)	1st	0.001	0.000–0.002
	2nd	0.000	0.000–0.001

*eGFR, estimated glomerular filtration rate; TP1NP, total procollagen type 1 N-terminal propeptide; PTH, parathyroid hormone; 25OHD, 25-hydroxyvitamin D*.

a *Linear regression analyses were conducted following these models: model 1 adjusted for age and sex; and model 2 adjusted for age, sex, waist circumference, hypertension, diabetes mellitus, systolic blood pressure, diastolic blood pressure, serum albumin, triglyceride, high-density lipoproteincholesterol, low-density lipoprotein cholesterol, and fasting blood glucose levels*;

b *Unstandardized β was provided and shown per 1-SD increment*;

c *P < 0.05*.

## Discussion

Previous studies exploring the association between mineral metabolism and renal function have been conducted in the CKD population, among general adults and within the elderly, and as the first time, all findings from the current study provided strong support for the association in Chinese centenarians. This cross-sectional study showed that the simple correlations and multivariate associations of serum phosphorus, osteocalcin, β-crosslaps, TP1NP, and PTH levels with eGFR levels were significant, but those of serum calcium and 25OHD levels with eGFR levels were not significant in Chinese centenarians.

The current study confirmed significant association between serum phosphate and renal function which was consistent with the results from previous studies (19, 20). CKD reduces renal phosphate excretion and increases serum phosphorus levels (21–23). Serum phosphate levels undergo diurnal changes across eGFR levels (23). Meanwhile, the current study determined significant association between serum PTH levels and renal function which corroborated previous work (7). Serum PTH levels have been realized to be different across eGFR levels (7). As a result of increased PTH production and parathyroid cell proliferation, elevated phosphate levels induce the release of PTH from parathyroid and an increase in serum PTH levels (24–26). Therapeutic strategy regulating mineral metabolism may be effective for preventing deterioration of renal function.

In terms of serum calcium and 25OHD levels, their correlations with renal function have been established in previous studies (7, 8, 27). However, serum calcium and 25OHD levels have been reminded to have no obvious change across eGFR levels (7). Serum 25OHD levels have been found to be not correlated with eGFR levels in another study (8). The current study did not obtain significant result about the association of serum calcium and 25OHD levels with renal function, which may be due to a delay of the change in both serum calcium and 25OHD levels. Serum calcium and 25OHD levels begin to decrease often at the late stages of CKD (eGFR <30 ml/min/1.73 m^2^), while eGFR levels were relatively maintained among these Chinese centenarians. Disordered phosphate and PTH metabolism generally precedes the change in serum calcium and 25OHD levels in the CKD population (7). Thus, serum phosphate and PTH levels could have a closer association with renal function than serum calcium and 25OHD levels (7).

In the CKD population, the prevalence of MBD has been reported to remain elevated with different pattern involving osteolysis and osteogenesis (28). Abnormal phosphate levels result in the change in osteolysis and osteogenesis, and disordered mineral metabolism aggravates the change in serum phosphate levels. When untreated for a long time, they may cause renal osteodystrophy and bone disease (29). Meanwhile, CKD-MBD has different types such as high and low turnover bone diseases (4). In the CKD population, MBD has complex pathogenesis, and secondary hyperparathyroidism is not its single pathogenesis (4). Secondary hyperparathyroidism, abnormal vitamin D metabolism, and other mechanisms play significant roles in the development of CKD-MBD. As the indices of osteolysis and osteogenesis, serum osteocalcin, β-crosslaps, and TP1NP levels were pinpointed to have a significant association with renal function in the current study. Previous studies have provided conflicting information on the association and their complex interrelationship within the body poses a real challenge explaining the association (30). However, the current study still obtained consistent results about the association of serum phosphate, osteocalcin, β-crosslaps, TP1NP, and PTH levels with eGFR levels in Chinese centenarians.

The current study had strength and limitation. The current study had a considerably population-based sample of centenarians, and these centenarian samples were extremely rare all over the world. Based on the current study with a cross-sectional design, further study is needed to obtain predictive models for multiple measures of mineral metabolism for renal function.

## Conclusion

Based on the data from CHCCS, this cross-sectional study supported that serum phosphorus, osteocalcin, β-crosslaps, TP1NP, and PTH levels were negatively associated with eGFR levels, and further demonstrated the significant association between multiple measures of mineral metabolism and renal function in Chinese centenarians. These findings could be helpful in promoting the development of medical tourism and health technology of Hainan, China.

## Data Availability Statement

The datasets generated for this study are available on request to the corresponding author.

## Ethics Statement

All procedures performed in studies involving human participants were in accordance with the ethical standards of Hainan branch of Chinese People's Liberation Army General Hospital (Sanya, Hainan; Number: 301hn11201601) and with the 1964 Helsinki declaration and its later amendments or comparable ethical standards. Informed consent was obtained from all participants included in the study.

## Author Contributions

SF, HY, YL, FLv, JD, FZ, FLu, YZ, and YY contributed to the study design, performed the data collection and analyses, and drafted the paper.

### Conflict of Interest

The authors declare that the research was conducted in the absence of any commercial or financial relationships that could be construed as a potential conflict of interest.
